# Early Detection of Skin Disorders and Diseases Using Radiometry

**DOI:** 10.3390/diagnostics12092117

**Published:** 2022-08-31

**Authors:** Amani Yousef Owda, Majdi Owda

**Affiliations:** 1 Department of Natural, Engineering and Technology Sciences, Arab American University, Ramallah P600, Palestine; 2 Faculty of Data Science, Arab American University, Ramallah P600, Palestine

**Keywords:** non-contact diagnoses, skin reflectance, basal cell carcinoma (BCC), squamous cell carcinoma (SCC), millimeter-wave, radiometry, diseased skin

## Abstract

Skin diseases and disorders have a significant impact on people’s health and quality of life. Current medical practice suggests different methodologies for detecting and diagnosing skin diseases and conditions. Most of these require medical tests, laboratory analyses, images, and healthcare professionals to assess the results. This consumes time, money, and effort, and the waiting time is stressful for the patient. Therefore, it is an essential requirement to develop a new automatic method for the non-invasive diagnosis of skin diseases and disorders without the need for healthcare professionals or being in a medical clinic. This research proposes millimeter-wave (MMW) radiometry as a non-contact sensor for the non-invasive diagnosis of skin diseases and conditions. Reflectance measurements performed using 90 GHz radiometry were conducted on two samples of participants; sample 1 consisted of 60 participants (30 males and 30 females) with healthy skin, and sample 2 contained 60 participants (30 males and 30 females) suffering from skin diseases and conditions, which were: basal cell carcinoma (BCC), squamous cell carcinoma (SCC), burn wounds, and eczema. Radiometric measurements show substantial differences in reflectance in the range of 0.02–0.27 between healthy and unhealthy regions of the skin on the same person. These results indicate that radiometry, as a non-contact sensor, can identify and distinguish between healthy and diseased regions of the skin. This indicates the potential of using radiometry as a non-invasive technique for the early detection of skin diseases and disorders.

## 1. Introduction

**Figure 1:**
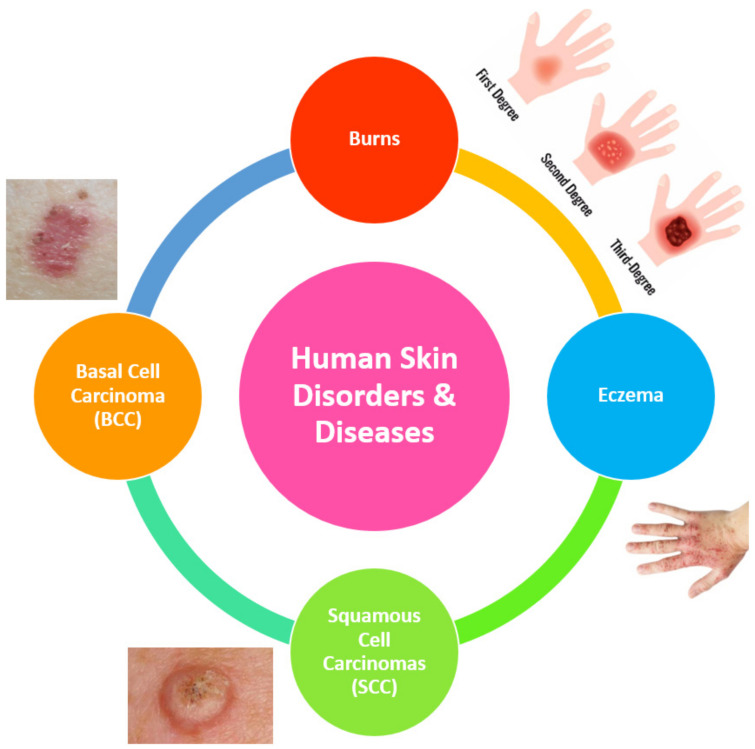
Skin conditions and diseases that might appear during the life cycle of a human. The images of the skin were obtained from open-access sources [8,9,10].

Human skin is the outer covering and multi-layered organ of the human body, having an average surface area of 2 m^2^ in adults [1,2,3]. Its main functions are protecting the body from external variations and organisms, providing the sense of touch, controlling the temperature of the body, and synthesizing vitamin D [1,4]. Human skin interacts directly with the environment (i.e., the sun, wind, heat, and radiation) [1]. Therefore, it suffers from multiple conditions and diseases, namely: dryness, burns, scars, skin cancers, and eczema, as illustrated in Figure 1, in addition to other factors, such as accidents, genetic defects, and increasing age from birth to death. These factors change the characteristics of the skin [5] as well as promote the development of skin disorders and diseases.

The World Health Organization (WHO) indicates a dramatic increase in the number of cases of skin cancers over the past few decades [6]. The organization reported around 3 million cases of non-melanoma skin cancers annually (i.e., basal cell carcinoma (BCC) and squamous cell carcinoma (SCC)) [6]. These cancers develop in non-covered regions of the body, such as the forearm, neck, face, and ears [4,6]. This is due to their direct exposure to ultraviolet radiation (UV) emitted by the sun and other artificial sources [6,7]. Early detection of skin cancer can reduce mortality and the cost of treatment, as well as significantly increase human survival.

Current medical practice for diagnosing non-melanoma skin cancers is based on a biopsy [8,11], which is the of removal of tissue to be tested under a microscope [12]. There are also other factors that help doctors in diagnosing skin cancer, such as the health status and age of the patient, signs on the skin, shapes of tumors, and symptoms [8,12]. This process requires specialist doctors for the assessment, cell removal, and prescription of treatments for patients. As a result, it consumes time and money and is painful to the patient. A non-invasive technique (e.g., millimeter-wave sensor) that can enable the automatic detection of skin cancers would be beneficial to humanity, healthcare professionals, sensor hardware engineers, medical device designers, and the healthcare technology community.

The millimeter-wave (MMW) band of the electromagnetic spectrum ranges from 30 GHz to 300 GHz [13,14,15]. In this frequency band, human skin reflectance and its signature are measured using either active sensing [16,17,18,19], which involves exposing the skin to non-ionizing MMW radiation [20,21], or passive sensing technology [22], which does not expose the skin to any type of man-made radiation [23]. The latter is the subject of this research.

Researchers in [16,17,24,25] used open-ended coaxial probes (i.e., active sensing) to measure the reflectance and dielectric properties of the skin. The measurements in [16,17,24,25] indicated variations in the dielectric properties and reflectivity of the skin from location to location and between dry and wet skin regions. This is due to variation in water content, which leads to changes in the reflectance of the skin [26]. The synthetic aperture radar (SAR) images of ex vivo samples of porcine skin in [18] showed a well-defined contrast in the reflectance between unburned and burne-damaged skin regions in the range of 0.05 to 0.1. These differences indicate the potential of using SAR images to detect burns under dressing materials. Researchers in [27,28] developed a model for estimating the reflectivity of healthy skin and skin with BCC. The results obtained from the model in [28] indicate differences in the dielectric properties of healthy skin and skin with BCC. This is due to the higher water content in BCC [29], which makes the refractive index of BCC higher than that of normal skin [27]. These results are not dissimilar from the measurements performed on healthy skin and skin with BCC using an open-ended coaxial probe in [30]. The study in [31] showed a well-defined contrast in reflectance between healthy skin and BCC. The importance of these results [31] is that the tumor margin is made distinguishable and clear. This will help doctors to identify the margin of the tumor. These results are supported by other studies conducted on normal and wet skin in [25,32]. The results obtained in [25,32] indicated higher dielectric properties and reflectance of wet skin (higher water content) compared with normal skin (lower water content). For burn-damaged skin, reflectivity measurements were performed on ex vivo porcine skin samples in [33] using an open-ended probe. The measurements showed lower reflectance of burn-damaged skin compared to normal skin. This is because the burning process evaporates water and makes the skin less reflective [34]. However, opposite results were obtained in an in vivo study conducted on human subjects using an open-ended coaxial probe, reported in [25]. This is because the burning process produces blood and exudates and makes the burned region of the skin more reflective compared with unburned skin [34]. However, an open-ended coaxial probe was used in many studies for the non-invasive diagnosis of skin diseases; the main challenges of using probes are their sensitivity to alignments as well as the need to provide constant pressure and be in direct contact with the target area of the skin [25,35,36]. These limitations make the probe unsuitable for measuring the reflectance of unhealthy regions of skin suffering from burns, injuries, and malignant lesions.

Millimeter-wave radiometry (i.e., passive sensor) was used to measure the signature of the skin (i.e., emissivity and reflectance) for the purposes of security screening [37] and medical applications [38]. Radiometric measurements in [15] showed differences in the mean emissivity values between normal and burn-damaged skin of chicken samples. These measurements indicated the potential of using radiometry for the non-invasive diagnosis of burn-damaged skin. The emissivity measurements in [22] indicated that the signature of the skin is dominated by the skin thickness, water content, age, and body mass index of healthy participants. The half-space electromagnetic model developed in [14] showed how the emissivity of the skin varies with frequency and estimated the mean emissivity values of healthy skin and diseased skin based on the relative complex permittivity data available in the open literature. The radiometric measurements in [39] indicated variation in the skin signature with physical activity and the hydration level of the skin (sweating). The reflectance measurements in [26] indicated variation in skin reflectance from location to location and between dry and wet skin regions. Further, the measurements indicated that human skin reflectance over the MMW band is dominated by skin thickness and water content [26]. These measurements [26] were aimed at assessing the significance level of differences in the reflectance between different regions of the human body, whereas the reflectance measurements in [40] investigated possible similarities in signatures between human skin and porcine skin. The reflectance measurements in this paper involve measurements in healthy and unhealthy groups of participants using radiometry for the first time; the unhealthy group consisted of participants with burns, non-melanoma skin cancers, and eczema. This research aimed to introduce radiometry as a non-contact sensor for detecting skin diseases and disorders without needing to be in a healthcare clinic and without the need for healthcare professionals or contact with the skin.

This section presents the background and reviews the state of the art of millimeter-wave technology in the non-invasive diagnosis of diseased skin; the following sections of the paper are structured as follows: Section 2 presents the experimental setup and the methodology used to measure the reflectance of healthy and unhealthy skin, and Section 3 shows the reflectance measurements for healthy and unhealthy skin regions. Section 4 discusses the main outcomes, and finally, Section 5 provides conclusions and motivations for future research directions.

## 2. Materials and Methods

This section presents the methodology used to measure the reflectance of human skin using a non-contact sensor (radiometry). The measurements were conducted in two groups of participants; group 1 included healthy participants, and group 2 comprised participants with eczema, malignancies, and burn wounds. Detailed information about the two groups of participants is given in Section 2.1. The materials and methods used to establish the experimental setup and conduct the measurements are discussed in Section 2.2, Section 2.3 and Section 2.4.

### 2.1. Participants

The reflectance measurements in this research were applied to healthy and unhealthy skin. Detailed descriptions of the two groups of participants are provided in the following sections.

#### 2.1.1. Participants with Healthy Skin

The measurements of healthy skin were conducted on 30 males and 30 females with no history of skin diseases or conditions. The participants’ ages ranged from 20 to 70 years. The means and standard deviations were 1.63 ± 0.1 m for height and 70.1 ± 11.54 kg for mass. The ethics application for this research was reviewed and approved by the ethics committee of Manchester Metropolitan University (MMU) under ethics reference number SE1617114C. A written consent form was signed and approved by each participant before conducting the measurements.

#### 2.1.2. Participants with Skin Conditions or Diseases

Sixty participants with different types of skin conditions and diseases in both genders (thirty males and thirty females) were recruited and measured in this research. The participants were divided into six groups, with each group containing 10 participants (5 males and 5 females): (1) participants with first-degree burns, (2) participants with second-degree burns, (3) participants with third-degree burns, (4) participants with skin SCC, (5) participants with BCC, and (6) participants with skin with eczema. The participants’ ages ranged from 20 to 70 years. The means and standard deviations of the height and weight of participants were 1.61 ± 0.085 m and 68.1 ± 8.75 kg, respectively. The ethics application for this research was reviewed and approved by the ethics committee of Manchester Metropolitan University (MMU) under ethics reference number SE1617114C. A written consent form was signed by each participant before conducting the measurements.

### 2.2. Measurement Locations

The reflectance measurements of healthy skin were performed on nine locations: (1) the palm of the hand, (2) the back of the hand, (3) the side of the inner wrist, (4) the middle of the inner wrist, (5) the outer wrist, (6) the dorsal surface, (7) the volar side of the hand, (8) the elbow, and (9) the finger region. For unhealthy participants, the measurements were performed on an unhealthy skin region (i.e., burns, eczema, malignancy, and basal cell carcinoma). Then, the measurements were compared with the reflectance of the closest region of healthy skin in the same participant. The measurement locations for unhealthy skin were chosen based on where the skin conditions or diseases appeared and where participants felt comfortable performing the measurements, such as the forearm, leg, neck, and face regions.

### 2.3. Experimental Setup

**Figure 2:**
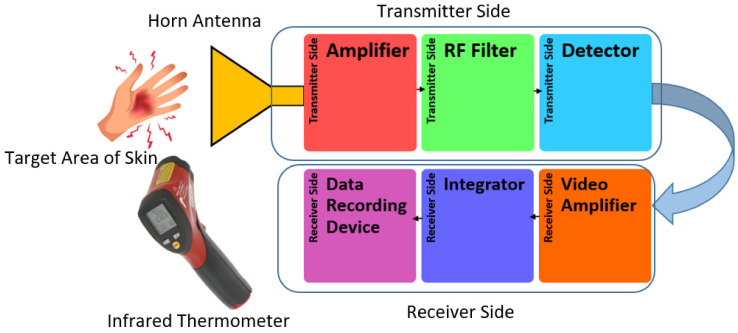
Experimental setup for the W-band radiometer used to measure the reflectance of healthy and unhealthy skin regions at a center frequency of 90 GHz.

A radiometer with a center frequency of 90 GHz and a bandwidth of 20 GHz (type: direct detection; manufacturer: Digital Barriers, Abingdon, UK) was used to measure the skin reflectance. The reflectance measurements in this research were applied to healthy and unhealthy skin. The radiometer consisted of two parts, namely: the transmitter and receiver sides, as described in Figure 2. The transmitter side has three main components: an amplifier, radio frequency (RF) filter, and detector. The receiver side consists of a video amplifier, integrator, and data recording device.

The horn antenna had a center frequency of 90 GHz and a bandwidth of 20 GHz (model number: AS4341; Atlan TecRF, Essex, UK). The antenna had a rectangular aperture of 30 mm × 25 mm and a nominal gain of 20 dB. During the experiment, the horn antenna was fixed to measure emissions from the target area of the skin and from hot and cold calibration sources. The voltage level of the emission from the target area of the skin was obtained from a distance of 5.0 cm from the skin sample and calibration sources. This distance was chosen as an optimal distance to minimize the chances of subjects accidentally touching and moving the measurement apparatus. A greater distance between the measured subject and the horn antenna would lead to measurements with a poorer spatial resolution [22].

The radiometer was calibrated using two pieces of carbon-loaded foam absorbers in liquid Nitrogen (cold calibration source with a temperature of 77 K) and ambient temperature (hot calibration source with a temperature of 293 K). The two pieces of carbon-loaded foam absorbers (model: Eccosorb AN-73; Laird, Shanghai, China) had a rectangular shape and dimensions: length = 180 mm, width = 140 mm, and thickness 10 mm. These dimensions were chosen because they are able to fill the horn antenna beam pattern and reduce the systematic uncertainty during the measurements.

An infrared thermometer (model number: N85FR; Maplin, Manchester, UK) was chosen to measure the temperatures of the target area of the skin and the calibration sources. This device was selected because the measurements were performed on healthy and unhealthy regions of the skin, so it is essential to obtain the temperature of the skin in a non-contact manner without touching the affected area of the skin.

After measuring the voltage levels and temperatures of the skin and calibration sources, the reflectance of the skin can be obtained using Equation (1). For further information about the derivation of Equation (1), readers can refer to [26].

(1) $$R=\frac{T_{S}\left(V_{H}-V_{C}\right)+T_{H}\left(V_{C}-V_{S}\right)+T_{C}\left(V_{S}-V_{H}\right)}{\left(T_{s}-T_{H}\right)\left(V_{H}-V_{C}\right)}\;$$

where $T_{s}$, $T_{H}$, and $T_{C}$ are the temperatures of the skin, hot source for calibration, and cold source for calibration, respectively. $V_{S},$ $V_{H}$ and $V_{C}$ are the voltage levels of the thermal emission (in mv) emitted from the skin and the hot and the cold calibration sources, respectively.

### 2.4. Methodology for Processing Data

**Figure 3:**
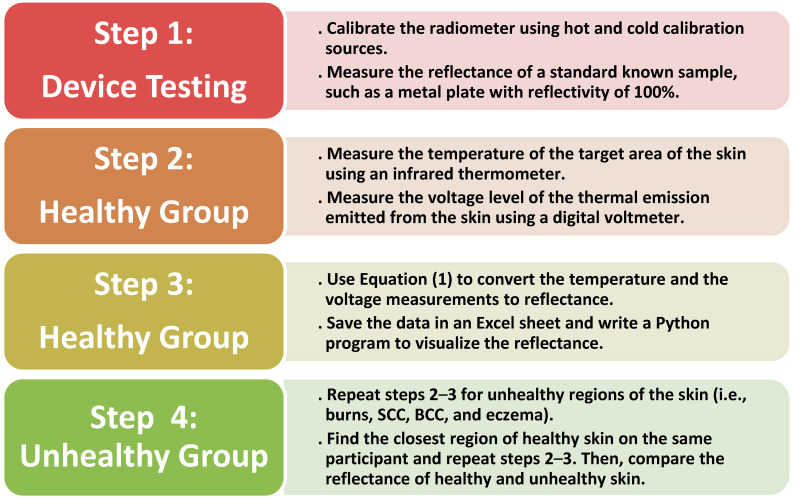
Methodology of collecting and processing the reflectance data for healthy and unhealthy regions of the skin.

The methodology for processing and collecting data is divided into four steps. Step 1 was device calibration and testing, in which two samples of foam absorbers (i.e., hot and cold calibration sources) were used to calibrate the radiometer. Then, standard samples with known reflectance, such as a metal plate, were measured. In step 2, the target area of the skin was located 5.0 cm from the horn antenna of the radiometer. This distance was chosen as an optimal distance to minimize the chances of subjects accidentally touching and moving the measurement apparatus. Then, an infrared thermometer and digital voltmeter were used to measure the temperature and voltage level of the thermal emission, respectively. In step 3, Equation (1) was used to convert the temperature and voltage measurements into reflectance. All data were saved as Excel files, and Python software was used to process and visualize the data. In step 4, a similar methodology was applied for unhealthy skin regions, except for conducting measurements on both unhealthy skin and the closest healthy skin region on the same person. Figure 3 summarizes the methodology used in this research.

## 3. Results

This section presents the reflectance measurements obtained from healthy skin and unhealthy skin.

### 3.1. Healthy Skin

**Figure 4:**
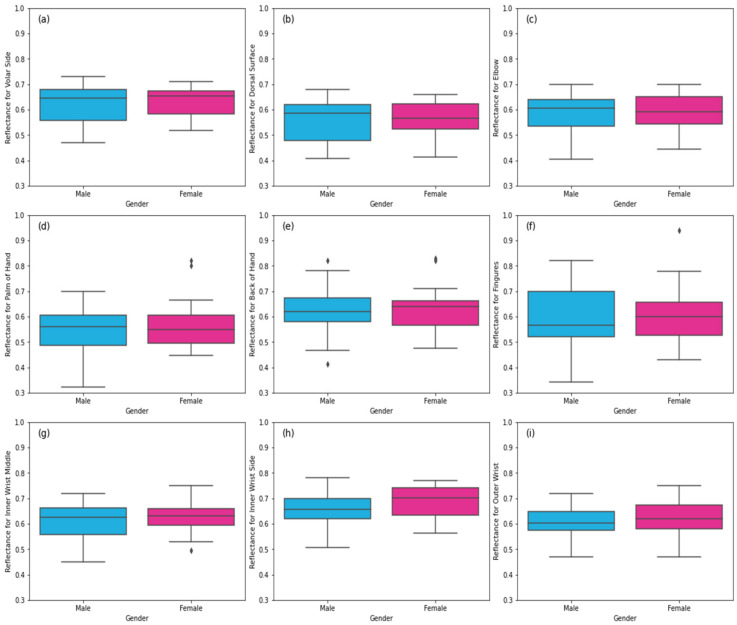
Reflectance measurements of healthy skin regions: (**a**) the volar side of the hand, (**b**) the dorsal surface of the hand, (**c**) the elbow, (**d**) the palm of the hand, (**e**) the back of the hand, (**f**) the finger region, (**g**) the inner wrist middle region, (**h**) the inner wrist side region, and (**i**) the outer wrist region. The symbol (◊) represents the outliers.

This section presents reflectance measurements of healthy skin regions. The reflectance measurements of a sample of 60 healthy participants (30 males and 30 females) in Figure 4 show variations in reflectance between males and females and also from location to location. The importance of having these measurements is that they identify the range of reflectance in healthy skin regions. This will help in identifying anomalies that might indicate diseased skin or skin disorders. An example of this is the outlier in Figure 4g, which shows a reflectance value of 0.449 for the inner wrist region. This value is from a 70-year-old female participant with no history of skin diseases. However, she suffered from dehydration due to loss of the sensation of thirst as a result of age [41]. Other outliers in Figure 4d–f are related to the weight of the participants; the researchers in [42,43] indicated that body mass index affected skin thickness. Therefore, participants who are overweight have thicker skin, which means that the blood vessels are far from the surface of the skin, and this results in lower reflectance. In contrast, participants who are underweight have thinner skin and higher reflectance as a result of blood vessels being close to the skin surface.

### 3.2. Unhealthy Skin

This section presents reflectance measurements performed on burned skin regions, skin with SCC, skin with BCC, and skin with eczema.

#### 3.2.1. Skin with First-Degree Burns

**Figure 5:**
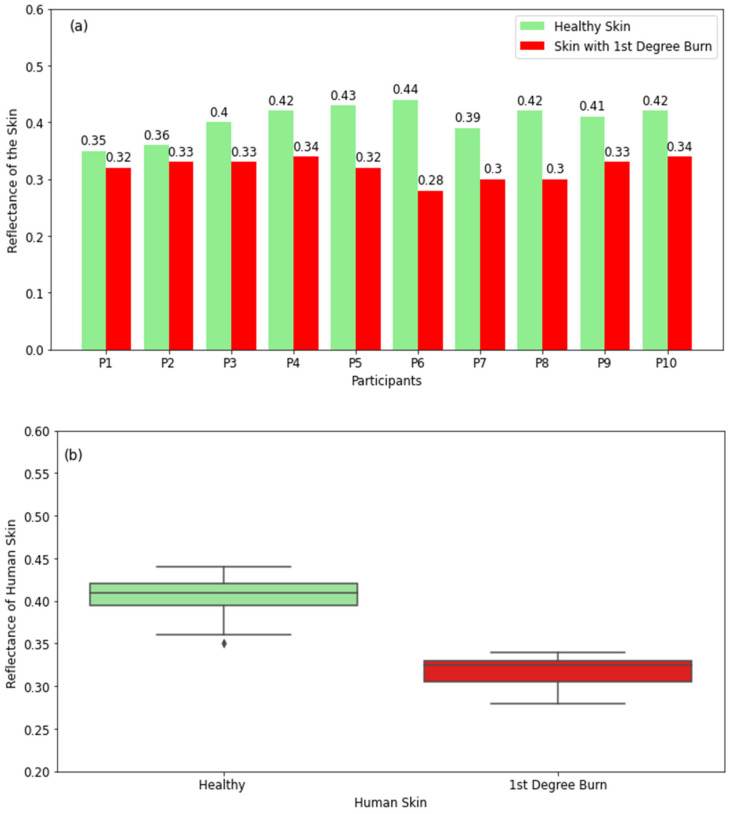
Reflectance measurements in a sample of 10 participants with 1st-degree burns compared with the closest region of unburned (or healthy) skin on the same participant (**a**). The boxplot shows the range of the reflectance of healthy skin and 1st-degree-burned skin (**b**). The symbol (◊) represents the outliers.

The reflectance measurements in Figure 5a show that healthy skin has higher reflectance compared with burned skin. The differences in reflectance between healthy and burned regions of the skin for all participants are found to be in the range of 0.03 to 0.16. The boxplots in Figure 5b provide a five-point summary of the reflectance of healthy and burned regions of the skin. The boxplot for healthy skin shows that the reflectance varies in the range of 0.36 (minimum value) to 0.44 (maximum value) with a median value of 0.415, whereas the range for burned skin is 0.28–0.34 with a median value of 0.325. These measurements confirm that there are substantial differences in the reflectance of healthy and burned regions of the skin. This indicates that radiometry can distinguish between unburned and burned skin regions.

#### 3.2.2. Skin with Second-Degree Burns

**Figure 6:**
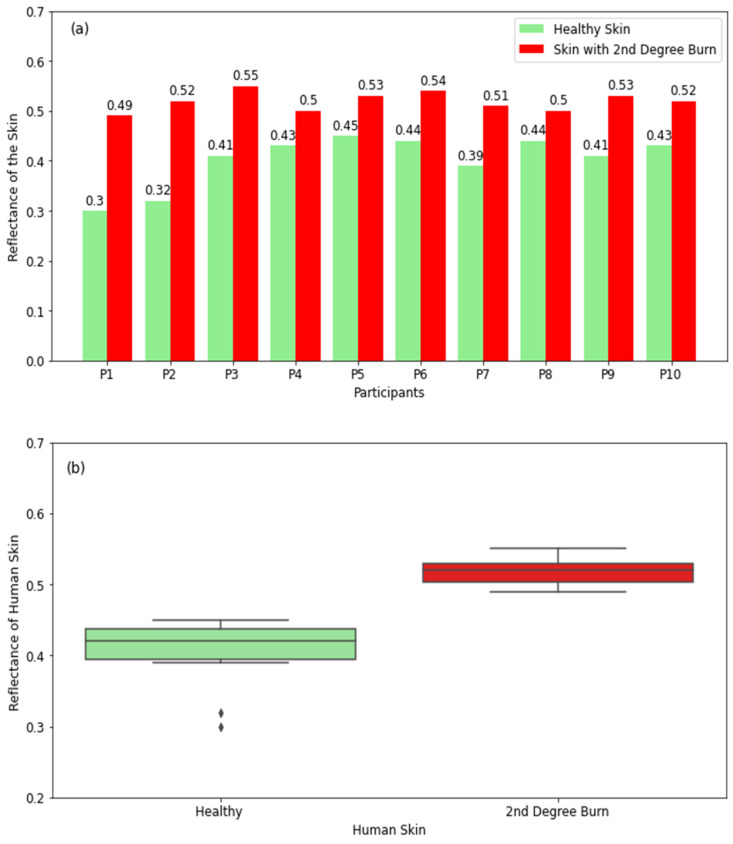
Reflectance measurements in a sample of 10 participants with 2nd-degree burns compared with the closest region of unburned skin on the same participant (**a**). The boxplot shows the range of the reflectance of healthy skin and 2nd-degree-burned skin (**b**). The symbol (◊) represents the outliers.

The experimental measurements in Figure 6a show that healthy skin has lower reflectance compared with skin with second-degree burns. This is due to the nature of second-degree burns, which produce wounds and exudates around the burned region, and as a result, this makes the burned skin more reflective compared with unburned skin. The differences in reflectance between healthy and burned regions of the skin for all participants are found to be in the range of 0.06 to 0.19. The boxplot for healthy skin in Figure 6b shows that the reflectance varies in the range of 0.39 (minimum value) to 0.45 (maximum value) with a median value of 0.42, whereas the range for burned skin is 0.49–0.55 with a median value of 0.52. The measurements indicate well-defined differences in reflectance between burned and unburned regions of the skin, and these differences are affected by the exudates and the severity of the burn.

#### 3.2.3. Skin with Third-Degree Burns

**Figure 7:**
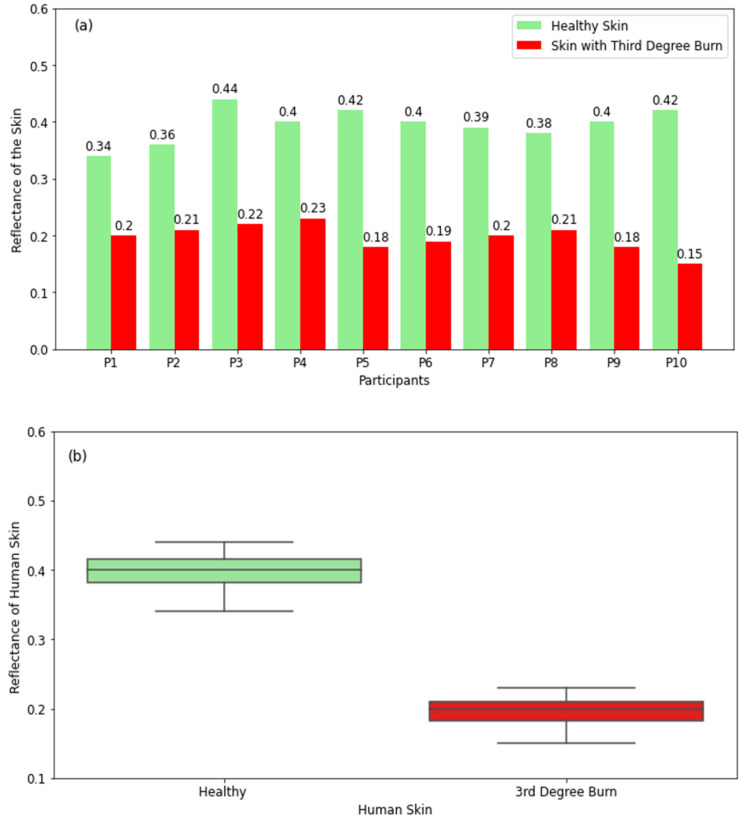
Reflectance measurements in a sample of 10 participants with 3rd-degree burns compared with the closest region of unburned skin on the same participant (**a**). The boxplot shows the range of the reflectance of healthy skin and 3rd-degree-burned skin (**b**).

The radiometric measurements in Figure 7a show that healthy skin has higher reflectance compared with skin with third-degree burns. This is because the full-thickness burn damages all skin layers as well as sensation and nerve endings. This makes the burned region of the skin very dry, and this results in lower reflectance. The differences in reflectance between healthy and burned regions of the skin for all participants are found to be in the range of 0.14 to 0.27. These differences are higher than those for first- and second-degree burns. These findings indicate that differences in reflectance depend on the severity of the burns. This suggests that radiometry can be used as a non-contact sensor to distinguish between different degrees of burns. The boxplot for healthy skin in Figure 7b shows that the reflectance varies in the range of 0.34 (minimum value) to 0.44 (maximum value) with a median value of 0.4, whereas the range for burned skin is 0.14–0.27 with a median value of 0.2. The measurements indicate well-defined differences in reflectance between burned and unburned regions of the skin.

#### 3.2.4. Skin with Squamous Cell Carcinoma (SCC)

**Figure 8:**
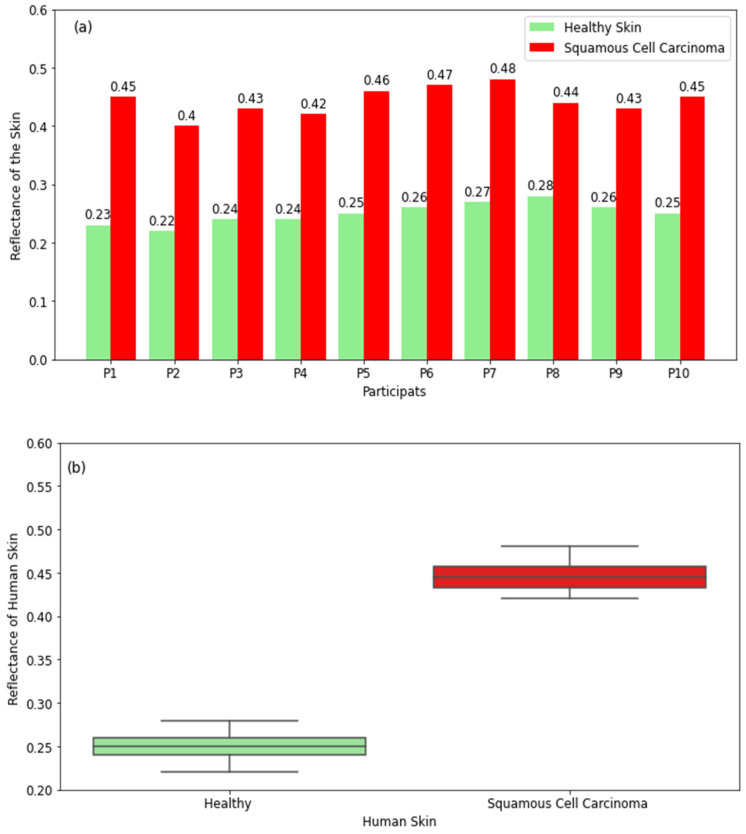
Reflectance measurements in a sample of 10 participants with squamous cell carcinoma compared with the closest region of healthy skin on the same participant (**a**). The boxplot shows the range in the reflectance of healthy skin and squamous cell carcinoma (**b**).

The experimental measurements of healthy skin and skin with squamous cell carcinoma in Figure 8a show that skin with squamous cell carcinoma has higher reflectance compared with healthy skin for all participants. This is due to the higher water content in squamous cell carcinoma [29,31], which makes the tumors more reflective compared with normal skin. The differences in reflectance between healthy skin and squamous cell carcinoma are in the range of 0.16 to 0.22. The boxplot for healthy skin in Figure 8b shows that the reflectance varies in the range of 0.22 (minimum value) to 0.28 (maximum value) with a median value of 0.25, whereas the range for squamous cell carcinoma is 0.42–0.48 with a median value of 0.445. The measurements indicate well-defined differences in reflectance between healthy skin and squamous cell carcinoma.

#### 3.2.5. Skin with Basal Cell Carcinoma (BCC)

**Figure 9:**
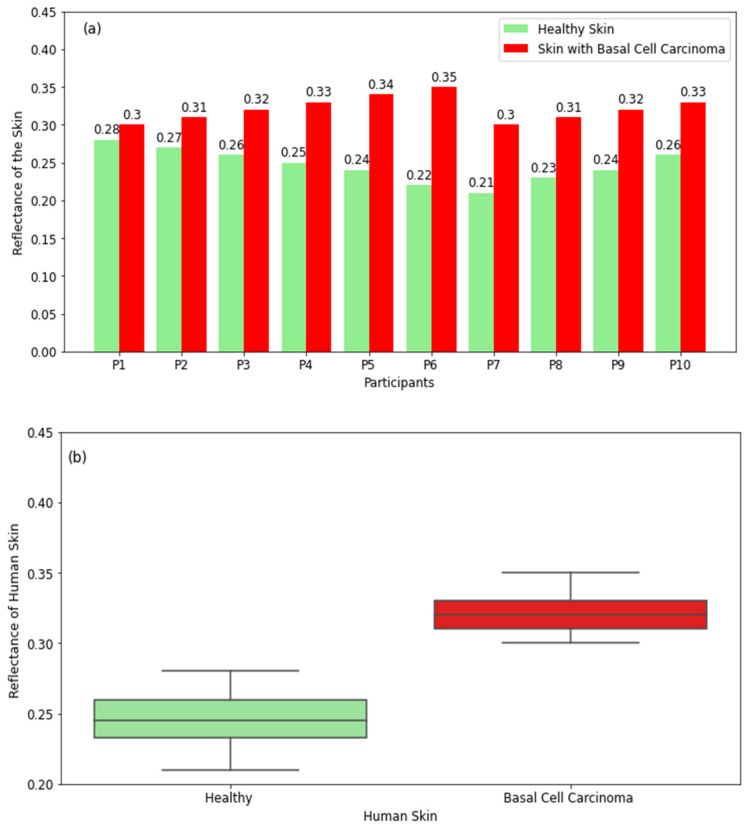
Reflectance measurements in a sample of 10 participants with basal cell carcinoma compared with the closest region of healthy skin on the same participant (**a**). The boxplot shows the range of the reflectance of healthy skin and basal cell carcinoma (**b**).

The reflectance measurements of healthy skin and skin with basal cell carcinoma in Figure 9a show that skin with basal cell carcinoma has higher reflectance compared with healthy skin for all participants. This is due to the higher water content in basal cell carcinoma [29,31]. The differences in reflectance between healthy skin and basal cell carcinoma are in the range of 0.02 to 0.13. The boxplot for healthy skin in Figure 9b shows that the reflectance varies in the range of 0.21 (minimum value) to 0.28 (maximum value) with a median value of 0.245, whereas the range for basal cell carcinoma is 0.3–0.35 with a median value of 0.32. The measurements indicate that radiometry can distinguish between healthy skin and skin with basal cell carcinoma.

#### 3.2.6. Skin with Eczema

**Figure 10:**
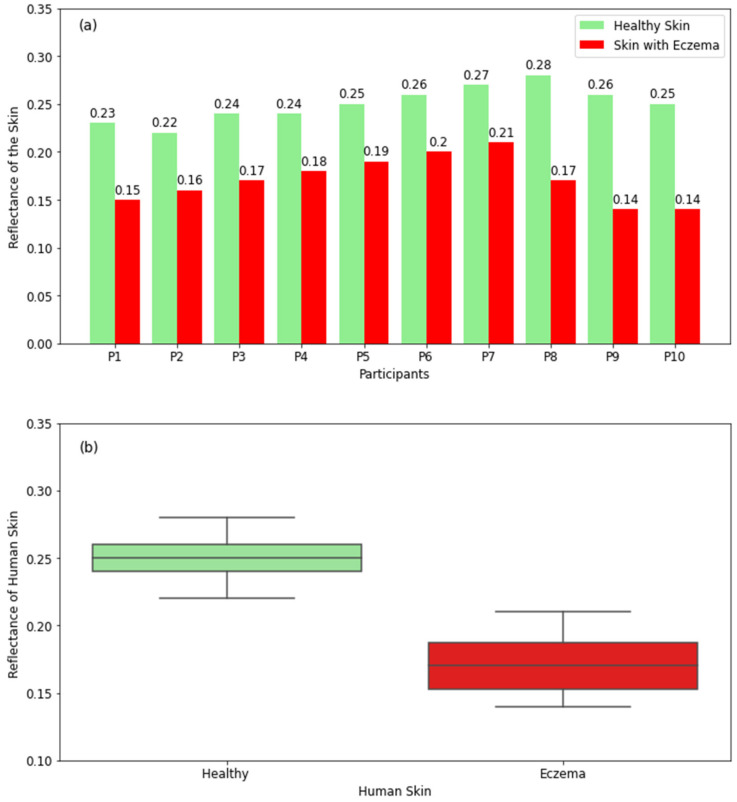
Reflectance measurements in a sample of 10 participants having eczema compared with the closest region of healthy skin on the same participant (**a**). The boxplot shows the range of the reflectance of healthy skin and eczema (**b**).

The experimental measurements for healthy skin and skin with eczema in Figure 10a show that healthy skin has higher reflectance than that of skin with eczema for all participants. This is because eczema makes the skin drier [8]. The differences in reflectance between healthy skin and skin with eczema are in the range of 0.06 to 0.12. The boxplot for healthy skin in Figure 10b shows that the reflectance varies in the range of 0.22 (minimum value) to 0.28 (maximum value) with a median value of 0.25, whereas the range for skin with eczema is 0.14–0.21 with a median value of 0.17. The measurements indicate that millimetric radiometry can be used as a non-invasive technique to detect skin conditions without contacting the skin.

## 4. Discussion

The reflectance measurements in this research confirm substantial differences in reflectance between healthy and unhealthy regions of the skin. This indicates that reflectance measurements can be a good indicator of the health status and disease risk of the skin. The key innovation is the introduction of millimeter-wave radiometry as a non-contact sensor for the non-invasive diagnosis of diseased skin and for the early detection of skin diseases and disorders.

The reflectance measurements of healthy skin regions in Figure 4 show the distribution of the reflectance at different locations on the hand for both genders. The radiometric measurements indicate substantial variations in reflectance from location to location and between males and females, as illustrated in Table 1. These variations are dominated by many factors: (1) skin thickness, (2) water content, (3) the chemistry of the dermis layer, (4) ethnicity, and (5) the body mass index [26].

The reflectance measurements of healthy and burned skin regions in Figure 5, Figure 6 and Figure 7 show well-defined differences in reflectance between healthy skin and burn-damaged skin. The measurements in Figure 5 and Figure 7 present lower reflectance for skin with first- and third-degree burns compared with healthy skin, whereas Figure 6 shows higher reflectance for second-degree burns. This is due to the presence of blood, lymph, and other exudates around second-degree burn wounds, which make the skin more reflective compared with unburned skin regions. Reflectance measurements indicate that differences in reflectance between healthy and burned regions of the skin are in the range of 0.03 to 0.27. These differences are found to be proportional to the degree and severity of the burn. This indicates the potential of using radiometry as a non-contact sensor to distinguish between different types of burns.

The experimental results for skin cancers in Figure 8 and Figure 9 show that squamous cell carcinoma and basal cell carcinoma have higher reflectance compared with healthy skin. Differences in reflectance between healthy and cancer regions are in the range of 0.02–0.22. These findings indicate the feasibility of using millimetric radiometry as a non-invasive sensor for the early detection of skin cancer.

The radiometric measurements in Figure 10 indicate that skin regions with atopic dermatitis (or eczema) have lower reflectance compared with normal regions. The differences in reflectance between normal skin and eczema are in the range of 0.06–0.12. This is because eczema is considered a dry-skin condition rather than a disease [8]. Eczema makes the skin sore, red, itchy, and dry [8] and, as a result, decreases the reflectance. Table 2 summarizes the main findings of healthy and unhealthy skin regions.

Radiometric measurements of human skin over the microwave and millimeter-wave bands are very sensitive to variations in water content and skin thickness [22,26]. Radiometric measurements of healthy skin [26] show variations in reflectance from person to person and from location to location in the range of 0.46 to 0.75. These measurements [26] aim to assess the significance level of differences in reflectance between different regions of the human body. These measurements are consistent with the reflectance measurements in this research. The passive images obtained from ex vivo porcine skin samples in [44] indicate a well-defined contrast in emissivity and reflectance in the range of 0.1–0.2 at 250 GHz. This contrast allows burns to be distinguishable and observable under dressing materials. Moreover, radiometric measurements obtained from chicken skin samples in [15] show that the mean difference in the emissivity of the skin (emissivity = 1-reflectance) increases with the degree of the burn in the range of 0.05–0.013. These results indicate the potential of using radiometry for the non-invasive diagnosis of diseased skin. The reflectance measurements in this paper involve measurements in healthy and unhealthy groups of participants using radiometry for the first time; the unhealthy group consisted of participants with burns, non-melanoma skin cancers, and eczema. The reflectance measurements in this paper indicate the feasibility of using radiometry as a non-contact sensor to detect skin diseases and disorders without needing to be in a healthcare clinic, without the need for healthcare professionals, and without needing to contact the skin.

## 5. Conclusions

This paper presents reflectance measurements performed on a sample of 120 participations (60 with healthy skin and 60 with unhealthy skin). The measurements were performed using millimeter-wave radiometry at a center frequency of 90 GHz. For the healthy skin group (30 males and 30 females), the measurements were conducted on nine locations on the hand: (1) the volar side of the hand, (2) the dorsal surface of the hand, (3) the elbow, (4) the palm of the hand, (5) the back of the hand, (6) the finger region, (7) the inner wrist (middle region), (8) the inner wrist (side region), and (9) the outer wrist region. The measurements show different ranges of reflectance for different locations and genders. In addition, exceptional data points (outliers) reflect the state of health and the weight of the participants. For the unhealthy skin group (30 males and 30 females), reflectance measurements were performed on diseased and healthy skin regions of the same participants. The measurements indicate substantial differences in reflectance of the skin between healthy and unhealthy regions, namely: burn wounds, skin with BCC, skin with SCC, and eczema. These findings indicate the potential for using radiometry to detect skin diseases, disorders, and conditions, as it provides a robust testing platform without touching the skin (non-contact mode). The next step of this research is to conduct more reflectance measurements in a more diverse population with healthy and diseased skin in both genders and then use these measurements to perform gender-based comparisons, as well as build machine learning algorithms for the automatic detection and prediction of human skin diseases and disorders.

## Figures and Tables

**Table 1:** Reflectance range for a sample of 60 healthy participants.

Skin Region	Reflectance Range (Females)	Reflectance Range (Males)
Volar Side	0.52–0.71	0.47–0.73
Dorsal Surface	0.41–0.66	0.41–0.68
Elbow	0.44–0.70	0.41–0.70
Palm of Hand	0.45–0.82	0.32–0.70
Back of Hand	0.48–0.83	0.41–0.82
Fingers	0.43–0.94	0.34–0.82
Inner Wrist, Middle	0.50–0.75	0.45–0.72
Inner Wrist, Side	0.56–0.77	0.51–0.78
Outer Wrist	0.47–0.75	0.47–0.72

**Table 2:** Reflectance range for a sample of 60 participants with skin diseases or conditions.

Skin Disease or Condition	Reflectance Range (Healthy Region)	Reflectance Range (Unhealthy Region)
First-Degree Burn	0.36–0.44	0.28–0.34
Second-Degree Burn	0.39–0.45	0.49–0.55
Third-Degree Burn	0.34–0.44	0.14–0.27
Skin with SCC	0.22–0.28	0.42–0.48
Skin with BCC	0.21–0.28	0.30–0.35
Skin with Eczema	0.22–0.28	0.14–0.21

## Data Availability

The data used in this paper were collected by the research team and can be shared with other researchers upon request.
